# Phenolic Compounds in Organic and Aqueous Extracts from *Acacia farnesiana* Pods Analyzed by ULPS-ESI-Q-oa/TOF-MS. In Vitro Antioxidant Activity and Anti-Inflammatory Response in CD-1 Mice

**DOI:** 10.3390/molecules23092386

**Published:** 2018-09-18

**Authors:** Delgadillo Puga Claudia, Cuchillo-Hilario Mario, Navarro Ocaña Arturo, Medina-Campos Omar Noel, Nieto Camacho Antonio, Ramírez Apan Teresa, López-Tecpoyotl Zenón Gerardo, Díaz Martínez Margarita, Álvarez-Izazaga Marsela Alejandra, Cruz Martínez Yessica Rosalina, Sánchez-Quezada Vanessa, Gómez Francisco Enrique, Torre-Villalvazo Iván, Furuzawa Carballeda Janette, Camacho-Corona María del Rayo, Pedraza-Chaverri José

**Affiliations:** 1 Departamento de Nutrición Animal Dr. Fernando Pérez-Gil Romo, Instituto Nacional de Ciencias Médicas y Nutrición Salvador Zubirán (INCMNSZ), CDMX 14080, Mexico; mario.cuchilloh@incmnsz.mx (C.-H.M.); maggiediazm@yahoo.com (D.M.M.); 2 Departamento de Alimentos y Biotecnología, Facultad de Química, Universidad Nacional Autónoma de México (UNAM), CDMX 04510, Mexico; arturono@unam.mx; 3 Facultad de Química, Departamento de Biología, Universidad Nacional Autónoma de México (UNAM), CDMX 04510, Mexico; omarnoelmedina@gmail.com (M.-C.O.N.); pedraza@unam.mx (P.-C.J.); 4 Instituto de Química, Universidad Nacional Autónoma de México (UNAM), CDMX 04510, Mexico; camanico2015@gmail.com (N.C.A.); mtrapan@yahoo.com.mx (R.A.T.); 5 Colegio de Posgraduados en Ciencias Agrícolas, Puebla 72760, Mexico; zgerardo@colpos.mx; 6 Departamento de Nutrición Aplicada y Educación Nutricional, Instituto Nacional de Ciencias Médicas y Nutrición Salvador Zubirán (INCMNSZ), CDMX 14080, Mexico; marselalejandra@yahoo.com; 7 Facultad de Química, Universidad Nacional Autónoma de México (UNAM), CDMX 04510, Mexico; yekann@hotmail.com; 8 Facultad de Química, Universidad Autónoma de Zacatecas, Zacatecas 98500, Mexico; vanelilla@gmail.com; 9 Departamento de Fisiología de la Nutrición, Instituto Nacional de Ciencias Médicas y Nutrición Salvador Zubirán (INCMNSZ), CDMX 14080, Mexico; egomezinn@gmail.com (G.F.E.); ivan.inn@gmail.com (T.-V.I.); 10 Departamento de Inmunología y Reumatología, Instituto Nacional de Ciencias Médicas y Nutrición Salvador Zubirán (INCMNSZ), CDMX 14080, Mexico; jfuruzawa@gmail.com; 11 Facultad de Ciencias Químicas, Universidad Autónoma de Nuevo León, Monterrey 64570, Mexico; maria.camachocn@uanl.edu.mx

**Keywords:** *Acacia farnesiana* pods, antioxidant and anti-inflammatory activities, bioactive compounds, polyphenols

## Abstract

Background: *Acacia farnesiana* (AF) pods have been traditionally used to treat dyspepsia, diarrhea and topically for dermal inflammation. Main objectives: (1) investigate the antioxidant activity and protection against oxidative-induced damage of six extracts from AF pods and (2) their capacity to curb the inflammation process as well as to down-regulate the pro-inflammatory mediators. Methods: Five organic extracts (chloroformic, hexanic, ketonic, methanolic, methanolic:aqueous and one aqueous extract) were obtained and analyzed by UPLC-ESI-Q-oa/TOF-MS. Antioxidant activity (DPPH•, ORAC and FRAP assays) and lipid peroxidation (TBARS assay) were performed. Assessment of anti-inflammatory properties was made by the ear edema induced model in CD-1 mice and MPO activity assay. Likewise, histological analysis, IL-1β, IL-6, IL-10, TNF-α, COX measurements plus nitrite and immunohistochemistry analysis were carried out. Results: Methyl gallate, gallic acid, galloyl glucose isomer 1, galloyl glucose isomer 2, galloyl glucose isomer 3, digalloyl glucose isomer 1, digalloyl glucose isomer 2, digalloyl glucose isomer 3, digalloyl glucose isomer 4, hydroxytyrosol acetate, quinic acid, and caffeoylmalic acid were identified. Both organic and aqueous extracts displayed antioxidant activity. All extracts exhibited a positive effect on the interleukins, COX and immunohistochemistry assays. Conclusion: All AF pod extracts can be effective as antioxidant and topical anti-inflammatory agents.

## 1. Introduction

The loss of the prooxidant-antioxidant balance in biological systems is responsible of many disorders resulting in oxidative damage [1]. Phytochemicals from plants help to maintain this equilibrium by enhancing the scavenging properties against reactive oxygen species (ROS) [2]. Also, inflammation is a biological process that responds to several internal and external agents and aggressors as infection or injuries which can turn into pathological and chronic stages affecting to the host [3]. Plant bioactive compounds are capable of down-regulating the activation of the mediators involved in the inflammatory cascade [4,5,6]. Bioactivity of phytochemicals to counteract inflammation is enhanced if plant bioactive compounds are tested as extracts [7,8,9]. Phenols are the frequent chemical group closely related to these effects [10,11]. *Acacia* genus contains phenols and some other phytochemicals like amines, alcaloids, essential oils, non-protein aminoacids, cumarines, diterpenes, fatty acids, triterpenes, phytosterols, saponines, flavonoids, gums and tannins. Bioactivity and health promoting properties of these phytochemicals include the abatement of some chronical disorders e.g., cancer, obesity, aging and diabetes [12]. In pods, leaves, stems, barks, flowers, and roots of *Acacia farnesiana* (L.) Willd (family: Fabeceae), commonly named as sweet acacia or Huizache, it has been described the occurrence of phytochemicals like albumin, gallic acid, glutelins, kaempferol, quercetin, methyl gallate, myricetin, naringenin, diosmetin, apigenin, catechin, ellagic acid, lupeol, α-amyrin, β-amyrin, β-sitosterol, ferulic and caffeic acids, among others. In this way, recently Hernández [13], isolated and described the structural characteristic of three new compounds in hexanic-cloroformic extract from AF pods: (3β,22*E*)-stigmasta-5,22-dien-3-ol, β-d-glucopyranoside, (3β,22*E*)-stigmasta-5,22-dien-3-yl and (2*S*)-2,3-dihydroxypropyl tetracosanoate. Some of which have been pointed out as protectors against ROS-induced damage [14]. Particularly, *A. farnesiana* (AF) pods have been previously recognized as resource of phytochemicals with effective protective properties against oxidative stress and antibacterial activity [13,15,16]. The consumption of AF as infusions and/or decoctions includes antidiarrheal, antispasmodic and anti-hyperglycemic uses and alleviation of dyspepsia [17,18].

The use of edible plants and their recommendations to alleviate health disorders, require exhaustive investigation to support the benefits or limitations. The aims of this study were: (1) to investigate the antioxidant activity and protection against oxidative induced damage of six extracts [chloroformic (CE), hexanic (HE), ketonic (KE), methanolic (ME), methanolic:aqueous (80:20 *v*/*v*); (MEAE) and one aqueous (AE)] from AF pod and (2) to test the same six extracts for their capacity to curb the inflammation process as well as to down-regulate the pro-inflammatory mediators.

## 2. Results

### 2.1. Total Polyphenols Content

Total polyphenols content from CE, HE, KE, ME, MAE and AE from AF pods were 506, 620, 594, 378, 399 and 565 mg of equivalents of gallic acid/100 g of dry matter, respectively. These results keep up a correspondence with non-polar and polar properties of solvents as well as their ability to extract phenolic units from AF pods.

### 2.2. AF Pod Extracts Analyzed by UPLC-ESI-Q-oa-/TOF-MS

In the six extracts, 12 phenolic compounds were identified by UPLC-ESI-Q-oa-/TOF-MS analysis which include: methyl gallate, gallic acid, galloyl glucose isomer 1, galloyl glucose isomer 2, galloyl glucose isomer 3, digalloyl glucose isomer 1, digalloyl glucose isomer 2, digalloyl glucose isomer 3, digalloyl glucose isomer 4, hydroxytyrosol acetate, quinic acid, and caffeoylmalic acid (Table 1). It is worth mentioning that the methyl gallate was identified in the six extracts and the gallic acid was found in the chloroformic, methanolic:aqueous and aqueous extracts (see all chromatographic spectra extracts in Figure S1).

### 2.3. The Effect of the Extracts on the Elimination of Radicals Is Concentration-Dependent

**Figure 1:**
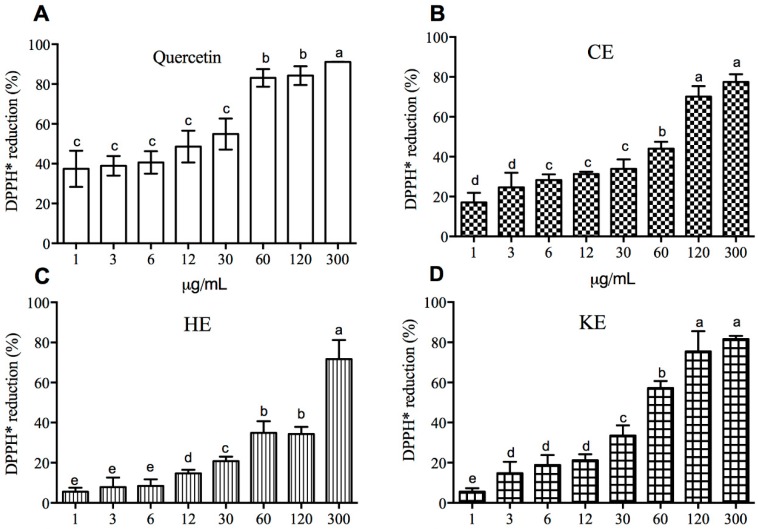

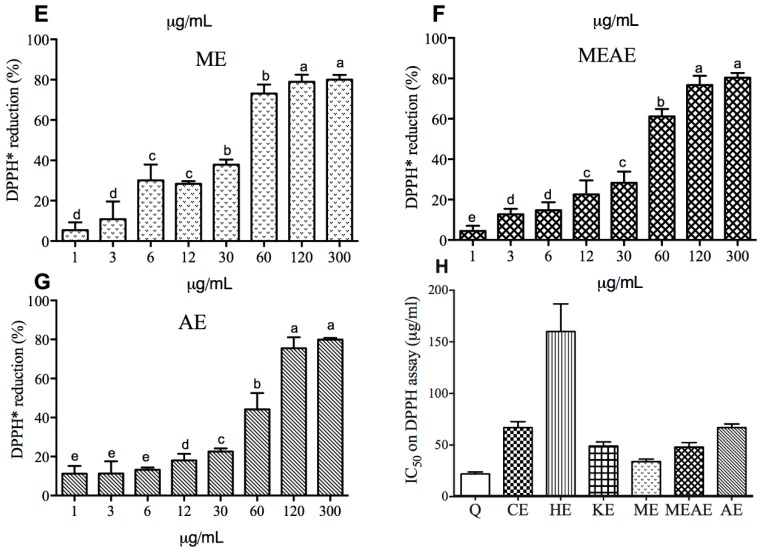
Scavenging capacity of the DPPH• radical (**A**–**G**) and inhibitory concentration 50% (IC_50_) values (**H**) of different extracts from *Acacia farnesiana* pods. The data are the mean with the standard deviation of three independent repetitions. CE = chloroformic extract; HE = hexanic extract; KE = ketonic extract; ME = methanolic extract; MEAE = methanolic:aqueous extract and AE = aqueous extract; Q = quercetin. ^a,b,c,d,e^ Different letters showed significant difference (*p* < 0.05) in the same extract at different concentrations (Kruskal–Wallis test).

As the concentration of the extracts increased, the radical scavenging capacity tended to increase as well (Figure 1A–G). No differences (*p* > 0.05) were observed among the six extracts when we compared the same concentrations (1.2–300 μg/mL) against quercetin. However, quercetin increased the scavenging capacity of DPPH• radical in the first five concentrations (from 1 to 30 μg/mL) and towards the end of the assay (Figure 1A). In contrast, HE, KE, ME and MEAE showed a much lower response to DPPH• discoloration at initial concentration and equated the other extracts at 300 μg/mL. In the same line, CE and AE responded to 120 and 300 μg/mL. However, below 60 μg/mL no favorable response was observed for the latest two extracts. In relation to IC_50_ value, quercetin showed the best result (22 μg/mL), while with KE, AE, CE and HE it was necessary to increase the concentration around to one, two, three and seven-fold more (respectively) than quercetin to scavenge 50 percent of DPPH• radical (Figure 1H).

### 2.4. Quantitative Antioxidant Activity

**Figure 2:**
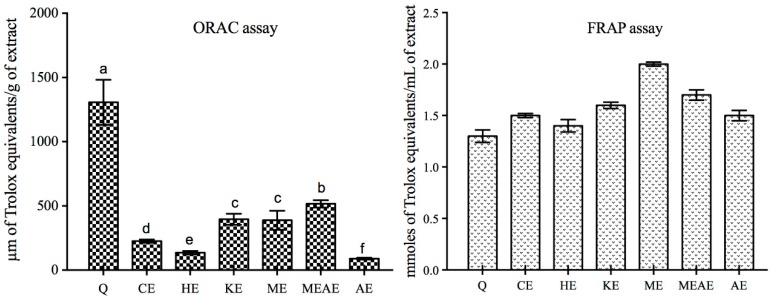
Total antioxidant activity of the extracts from *Acacia farnesiana* pods by oxygen radical absorbance capacity (ORAC) and ferric-reducing antioxidant power (FRAP) assays. Q = quercetin; CE = chloroformic extract; HE = hexanic extract; KE = ketonic extract; ME = methanolic extract; MEAE = methanolic:aqueous extract and AE = aqueous extract. ^a,b,c,d,e,f^ Different letters showed significant difference (*p* < 0.05) in the same extract at different concentrations (Kruskal–Wallis test).

ORAC values of the different extracts evaluated are show in Figure 2. All the extracts were less active than quercetin. Among all the extracts, MEAE showed the best performance (516 μmoles of Trolox equivalents/g of extract) followed by KE, ME, CE, HE and AE with 396, 388, 226, 136, and 89 μmoles of Trolox equivalents/g of extract, respectively. Differences in showed values were statistically significant. In marked contrast to ORAC, the extracts were more active than quercetin in the FRAP assay. In the FRAP assay, the first five tested concentrations (from 0.6 to 12 mmoles of Trolox equivalents/g of extract) were not different among all the extracts (data not showed). The depicted concentration is 30 mmoles of Trolox equivalents/g of extract; where, ME was the most efficient extract to reduce Fe^+3^ to Fe^+2^ with 2.0 mmoles of Trolox equivalents/g of extract, followed by MEAE, KE, CE, AE and HE, which needed 1.7, 1.6, 1.5, 1.5 and 1.4 mmoles of Trolox equivalents/g of extract, respectively. However, this is not statistically different (*p* > 0.05). Similarly, when we evaluated the six extracts at higher concentrations (60, 120 and 300 mmoles of Trolox equivalents/g of extract), no differences were observed. When we evaluated a particular extract at different concentration, we observed significant differences (*p* < 0.05). However, the highest concentration (300 mmoles of Trolox equivalents/g of extract) resulted in the best ferric-reducing antioxidant power. The values for this activity were: ME, 8.4; MEAE, 6.6; KE, 6.1; AE, 5.4; CE, 4.9 and HE, 3.4 μg Trolox equivalents/g, respectively. Regarding to FRAP, we observed IC_50_ values of 1.8, 1.5, 2.0, 2.6, 2.0 and 1.9 for CE, HE, KE, ME, MEAE and AE compared to 1.4 of quercetin.

**Figure 3:**
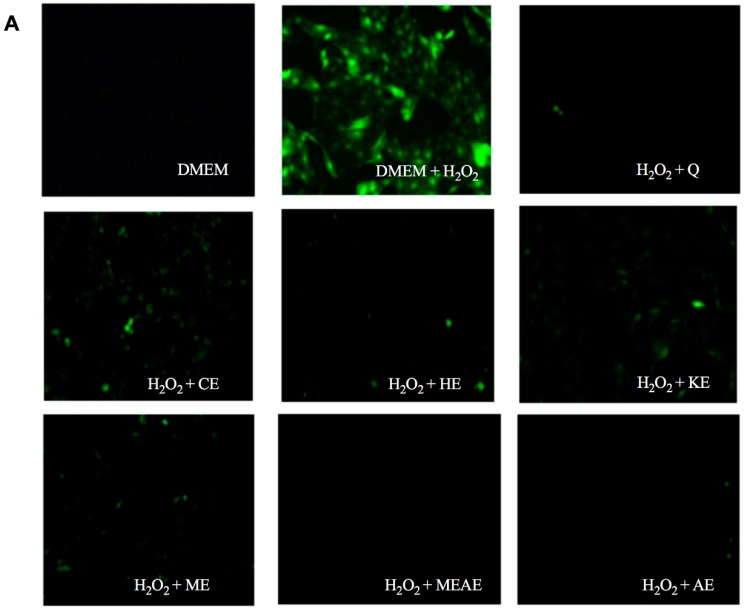

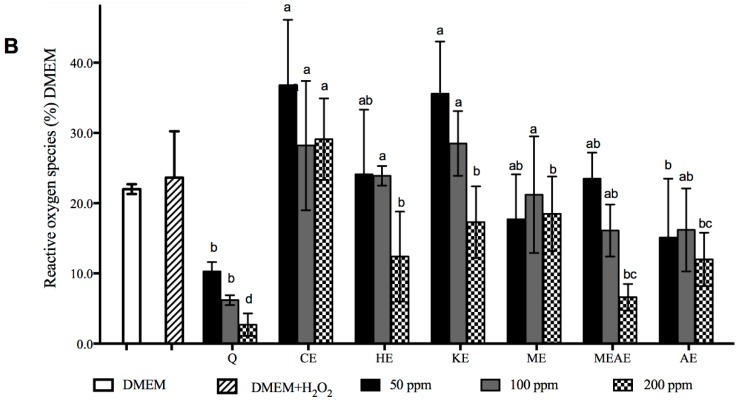
Representative images of reactive oxygen species (ROS) produced on porcine kidney cells exposed to H_2_O_2_ and to 200 ppm of extracts of *Acacia farnesiana* pods (**A**) and ROS quantification using 50, 100 and 200 ppm of extracts (**B**). H_2_O_2_ = hydrogen peroxide; Q = quercetin; CE = chloroformic extract; HE = hexanic extract; KE = ketonic extract; ME = methanolic extract; MEAE = methanolic:aqueous extract; AE = aqueous extract. ^a,b,c,d^ Different letters showed significant difference (*p* < 0.05) among extracts and queretin at the same concentration (Kruskal–Wallis test).

Figure 3 compares the quantitative and qualitative basis the protective effect of extracts. We made sure that extracts did not cause any pro-oxidative effect on cells at any of the tested concentrations. Figure 3A shows a picture selection of cell cultures treated with extracts at 200 ppm since they were the most representative to observe the contrasting effects of extracts over ROS production. An evident difference between cells treated with DMEM alone or with H_2_O_2_ was observed in the cellular lysis detected as brilliant green fluorescent spots. Quercetin showed the best protective capacity than extracts to inhibit the production of ROS (Figure 3B). In contrast, CE and KE demonstrated the poorest inhibition of ROS production.

### 2.5. Lipid Peroxidation Is Inhibited by All Extracts in a Dose-Dependent Manner

**Figure 4:**
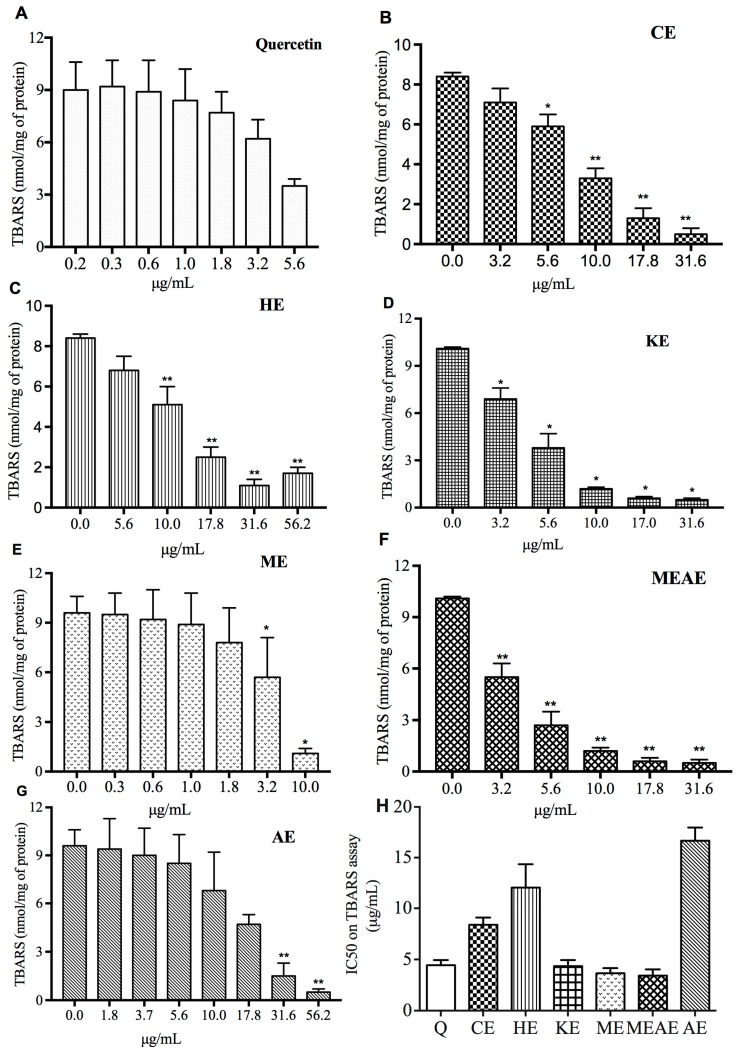
Inhibition of lipid peroxidation (**A**–**G**) and inhibitory concentration 50% (IC_50_) values (**H**) of the extracts from AF pods and standard quercetin, quantified by lipid peroxidation (TBARS) production. CE = chloroformic extract; HE = hexanic extract; KE = ketonic extract; ME = methanolic extract; MEAE = methanolic:aqueous extract and AE = aqueous extract. Q = quercetin. The data are the mean with the standard error of three independent repetitions. * *p* < 0.05 and ** *p* < 0.01 vs. control group.

Results of TBARS inhibition are shown in Figure 4. Inhibition of lipid peroxidation was observed in a dose-dependent manner in all extracts. Methanolic extract decreased TBARS production in 93% with the smallest concentration (10 μg/mL; equivalent to 1.0 nmol of TBARS/mg of protein). Moreover, 31.6 μg/mL equivalent to 0.5 nmol of TBARS/mg of protein of CE, KE and MEAE were needed to reach over 90% of TBARS inhibition, while AE and HE resulted in the same inhibition percentage with 56.2 μg/mL equivalent to 0.7 nmol of TBARS/mg of protein. In relation to quercetin, it is noteworthy that with only 5.6 μg/mL equivalent to 3.5 nmol of TBARS/mg of protein resulted in a TBARS inhibition over 60%. The 50% inhibitory concentration (IC_50_) values of the extracts on TBARS production are showed in Figure 4H. The IC_50_ value of KE, ME and MEAE not showed a statistical variation (*p* > 0.05) in respect to the standard quercetin. MEAE registered 3.4 nmol/mg, similar to ME; while CE, HE and AE IC_50_ were around to two, three and five-folds of that concentration and were less active than standard quercetin. Finally, KE and quercetin standard have similar values of IC_50_.

### 2.6. Acacia farnesiana Extracts Down Regulate Inflammation

**Figure 5:**
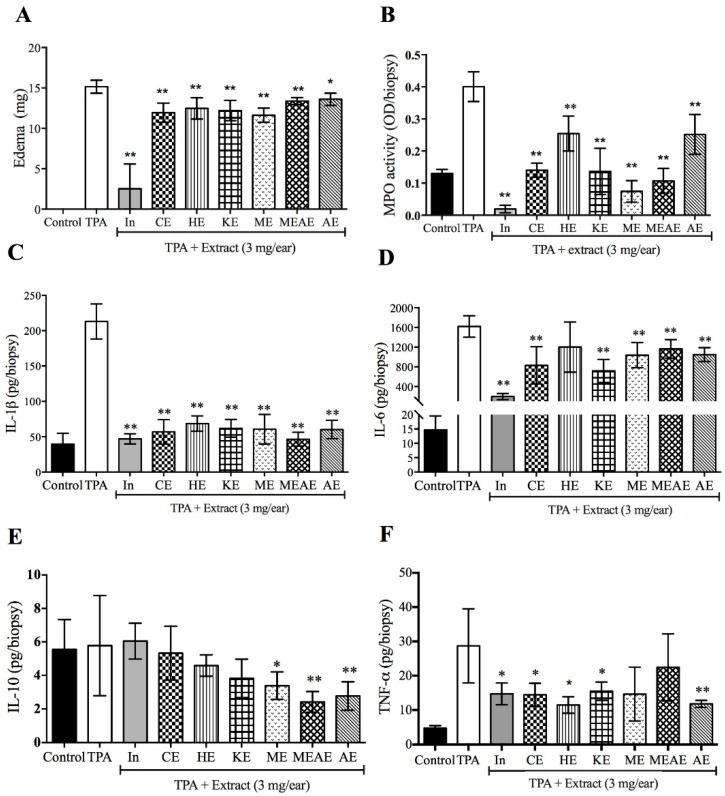
Effect of different extracts from *Acacia farnesiana* pods and the anti-inflammatory indomethacin (1 mg) on TPA-induced ear edema model. (**A**) ear edema measured at 4 h after TPA treatment and (**B**) oxidative enzyme myeloperoxidase (MPO) activity in supernatants of homogenates from TPA-treated ears. Levels of (**C**) interleukin-1β (**D**), interleukin-6 (**E**), interleukin-10 and (**F**) TNF-α in supernatants of homogenates from ears after treatment with different extracts from AF pods. TPA = 12-*O*-tetradecanoylphorbol acetate; In = indomethacin; CE = chloroformic extract; HE = hexanic extract; KE = ketonic extract; ME = methanolic extract; MEAE = methanolic:aqueous extract and AE = aqueous extract. Each bar represents the mean ± standard deviation (*n* = 6). * *p* < 0.05 and ** *p* < 0.001 with respect to TPA group.

In the ear edema model, all extracts diminished the ear tissue weight after TPA (12-*O*-tetradecanoylphorbol-13-acetate) application. However, the extracts were less effective than indomethacin to prevent edema (Figure 5A). Likewise, all extracts showed an inhibition on MPO and IL-1β production (Figure 5B,C). Similar results were observed by IL-6 (Figure 5D), where the production of this interleukin was inhibited by all the extracts (except by HE). For IL-10, TPA group showed comparable values with respect to indomethacin, CE, HE and KE treatments. In contrast, ME, MEAE and AE reduced the value of this anti-inflammatory cytokine (Figure 5E). TNF-α production were inhibited by all groups treated with AF extracts, except in the groups treated with ME and MEAE (Figure 5F).

### 2.7. Effect of Acacia farnesiana Extracts on Mice Ear Swelling

**Figure 6:**
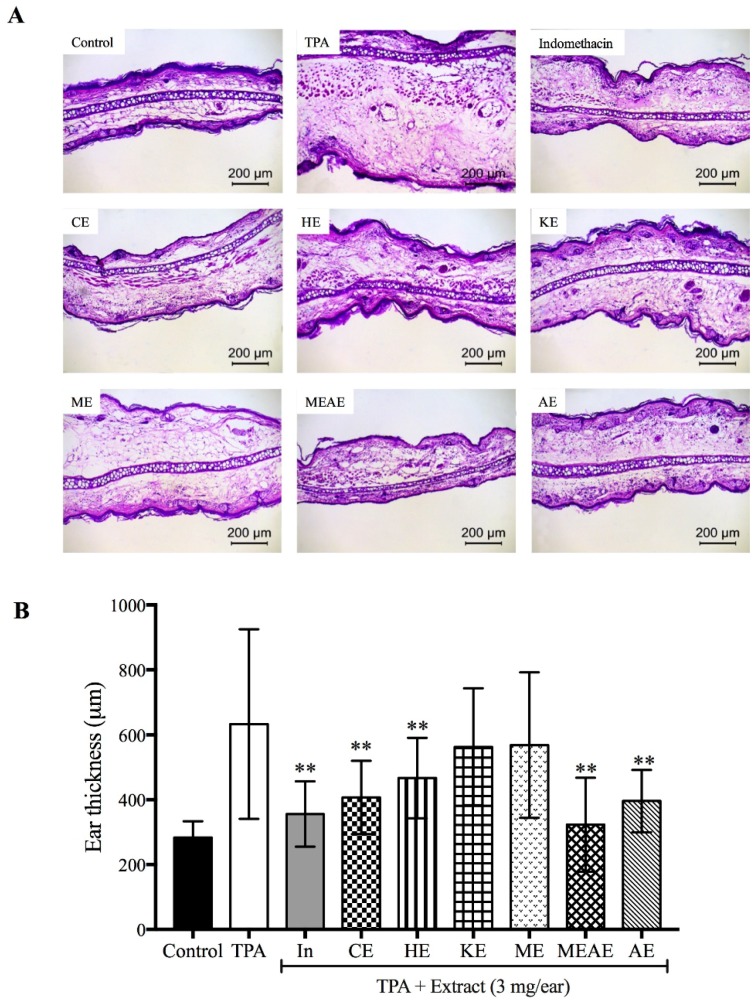
Histological features of mice ear tissue (**A**) stained with hematoxylin-eosin (200× magnification) on mice ear tissue thickness by the effect of different extracts of *Acacia farnesiana* pods on the TPA-induced ear edema model (**B**). TPA = 12-*O*-tetradecanoylphorbol acetate; In = indomethacin; CE = chloroformic extract; HE = hexanic extract; KE = ketonic extract; ME = methanolic extract; MEAE = methanolic:aqueous extract and AE = aqueous extract. Each bar represents the mean ± standard deviation (*n* = 6). ** *p* < 0.001 with respect to TPA group.

Figure 6 shows the mice ear tissue swelling as well as the ear thickness due to the effect of extracts on the TPA-induced ear edema model. All the evaluated extracts were different from control (*p* < 0.001). A morphological change due to the aggressive agent (TPA) resulted in a marked swelling process, sub-epidermal layer and spongiosis (Figure 6A). After TPA application, MEAE decrease the thickness of the mice ears almost at the same extent than indomethacin (323 and 356 µm; MEAE and indomethacin, respectively). In contrast KE and ME did not show a satisfactory reduction of the mice ears thickness in relation to control (Figure 6B).

### 2.8. Effect of Acacia farnesiana Extracts on TNF-α-Expressing Cells

**Figure 7:**
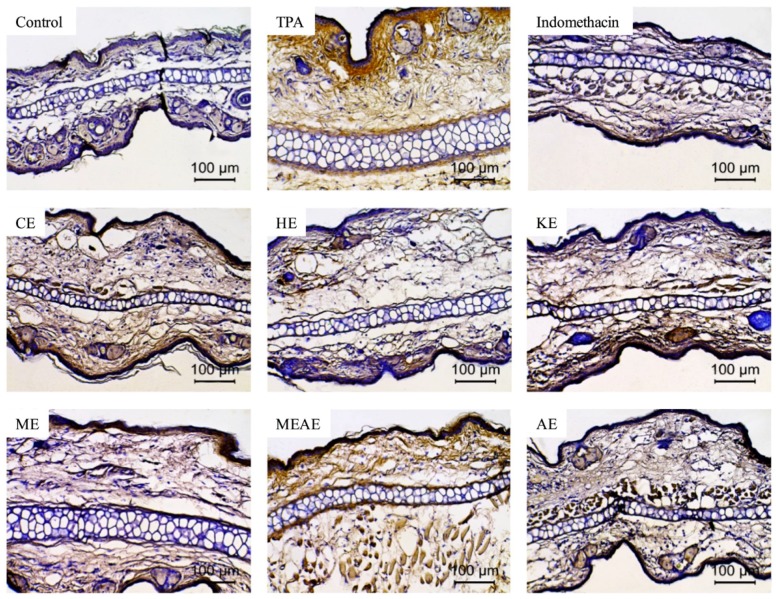
TNF-α expression (golden yellow color) in the mice ear cells due to the effect of different extracts of *Acacia farnesiana* (AF) pods (3 mg/ear) on the TPA-induced ear edema model (100× magnification). CE = chloroformic extract; HE = hexanic extract; KE = ketonic extract; ME = methanolic extract; MEAE = methanolic:aqueous extract and AE = aqueous extract.

Figure 7 shows the inflammation post treatment with different extracts of *Acacia farnesiana* (AF) pods on the TPA-induced ear edema model, by the expression of TNF-α by immunohistochemistry. While TPA induced conspicuous release of the pro-inflammatory mediator in the ears, treatment with CE, HE, KE, ME and AE resulted in an evident reduction in TNF-α production similar to indomethacin, while MEAE was the only that did not reduce of the TNF-α expression.

### 2.9. Effect of Acacia farnesiana Extracts on COX Activity, Cell Viability and Nitrite Production

**Figure 8:**
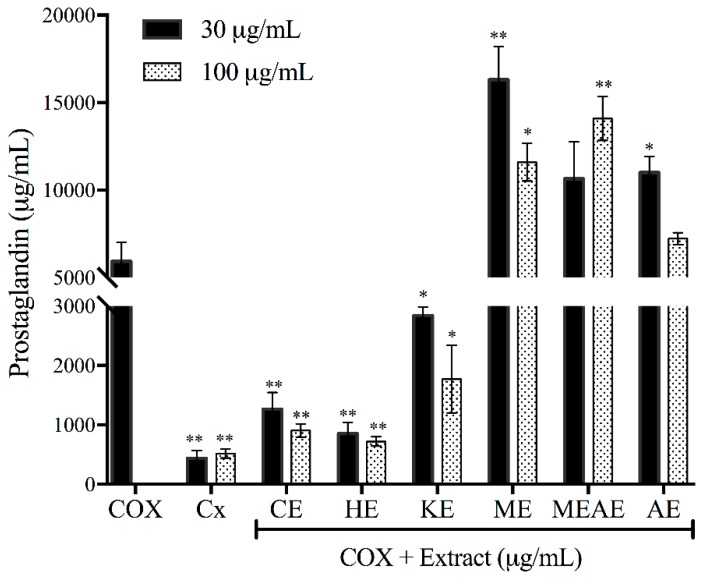
Effect of different *Acacia farnesiana* extracts on prostaglandin production. Cx = Celecoxib; CE = chloroformic extract; HE = hexanic extract; KE = ketonic extract; ME = methanolic extract; MEAE = methanolic:aqueous extract and AE = aqueous extract. The data are the mean with the standard deviation of three independent repetitions. Statistical difference was calculated in relation to COX at 30 and 100 ppm. * *p* < 0.05. ** *p* < 0.001.

**Figure 9:**
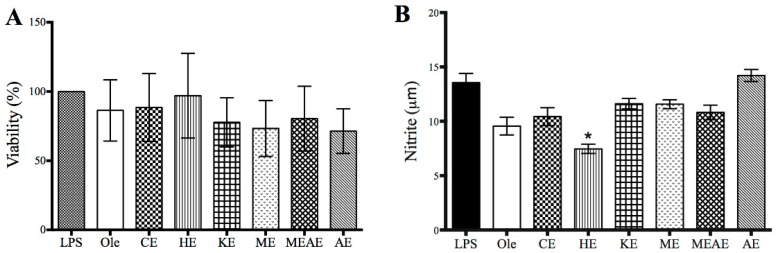
(**A**) Viability of RAW 264.7 cells treated with of lipopolysaccharides (LPS) of *E. coli* (1 µg/mL), oleanolic acid or different extracts (25 µg/mL) from *Acacia farnesiana* pods. (**B**) Nitrite concentration in supernatants of RAW 264.7 cells treated with LPS of *E. coli* (1 µg/mL), oleanolic acid (Ole) different extracts (25 µg/mL) from *Acacia farnesiana* (AF) pods. CE = chloroformic extract; HE = hexanic extract; KE = ketonic extract; ME = methanolic extract; MEAE = methanolic:aqueous extract and AE = aqueous extract. Each bar represents mean ± standard deviation (*n* = 6). * *p* < 0.05 between extracts respect to LPS.

The inhibitory activity of cyclooxygenase (COX) is shown in Figure 8. CE, HE and KE evaluated at 10 and 30 µg/mL were found to have inhibitory activity similar to control group (Celecoxib). In contrast ME, MEAE and AE demonstrated lower ability to inhibit the prostaglandin synthesis. The viability of RAW 264.7 cells treated with of lipopolysaccharides (LPS) of *E. coli* is shown in Figure 9A. The percentage of viability was over 70% in all groups evaluated and was similar to the control group (treated with oleanolic acid). Numerically, HE reported the highest value (96.9%) while AE the lowest percentage of viability (71.3%), however, no significant difference was found. With respect to the nitrite production (Figure 9B), HE showed the best inhibitory potential (7.4 µM). Moreover, nitrite production of cells treated with any of the left extracts (any but HE), were between oleic acid group (9.5 µM) and lipopolysaccharides group (13.5 µM) values.

## 3. Discussion

Salem et al. [27] analyzed *Acacia nilotica* pods extracts and isolated eight bioactive compounds, e.g., methyl gallate, gallic acid and 3 galloyl and digalloyl glucose isomers among others. In addition, with their results that confirmed anti-tumoral activity from pods extracts from this species, we have a coincidence of four of these compounds and confirmed its antioxidant and anti-inflammatory activity in the different extracts evaluated.

Total polyphenols content from CE, HE, KE, ME, MEAE and AE from AF pods were 506, 620, 594, 378, 399 and 565 equivalents of gallic acid, mg/100 g dry matter, respectively. Both results (yield and total polyphenols content) keep up a correspondence with non-polar and polar properties of solvents as well as their ability to extract phenolic units from AF pods.

The anti-inflammatory activity of these extracts against induced edema was confirmed when the ME was employed in rodent models (rat and mice). According to these results our findings with TLC and HPLC of HE and CE demonstrated the presence of both metabolites. Lin et al. [28] indicated that betulinic acid is another compound with potent anti-inflammatory and cytotoxic activity found in AF roots that might contribute to the beneficial properties of Acacia genus. Therefore, it is highly desirable to further investigate the identification and isolation of bioactive compounds in this species to characterize the phytochemicals responsible of the antioxidant and anti-inflammatory activities here described. Other authors indicated that betulinic acid also could have antioxidant activity [29], therefore it might contribute to the radical scavenging capacity of the chloroformic extract, where this metabolite was found. All extracts evaluated revealed antioxidant activity but with dissimilar responses among them. This observation is in line with the study of Delgadillo et al. [16] who evaluated Acacia pods and found an antioxidant activity of 95% with 200 μg/mL. In the present study, we observed a response near to 80% of antioxidant activity at 120 μg/mL. HE is the less polar extract and resulted as the less active on DPPH• extract evaluation. Though, ME and HE were able to remove lipidic compounds as triterpenoids with important scavenging capacity, HE was not capable to intensify the antioxidant activity until it was tested at a higher concentration. Karoune et al. [30] analyzed leaves and bark from *A. albida*. In that study, the IC_50_ results in DPPH• assay was similar between the two extracts obtained with ethanol (27 and 29 μg/mL). The IC_50_ in our study by DPPH• test resembled the ME with a value of 34 μg/mL. KE and MEAE were also similar to ME which were less effective than quercetin. Moreover, the use of other extractants was not as effective as ethanol employed in *A. albida*. Some other sources of variation among the results, apart from the polarity nature of solvents and the different vegetal parts in both studies, are possibly related to the different season of recollection, climate changes, distinct maturity stage and the different geographical sites of sampling. However, the results of DPPH• assay are similar to ORAC assay outcomes. The MEAE showed the best antioxidant activity among all extracts evaluated. It was 2.5 times less active than quercetin, while aqueous extract was close to 15 times less active than the standard, resulting in the least effective one. In marked contrast to ORAC test, the ME and MEAE were more active than the standard to reduced Fe^+3^ to Fe^+2^ in the FRAP assay. Likely, hydroxycinnamic acids and flavonoids are in large part responsible of the radical scavenging activity, since caffeic, cinnamic, chlorogenic, and gallic acids as well as catechin and epicatechin are recognized as potent radical stabilizers [1,14,15].

Regarding to the protective activity of the extracts against harmful effects of H_2_O_2_ to cells, at 200 ppm AF extract inhibited the production of reactive oxygen species better than 100 and 50 ppm. MEAE was the most active extract to protect kidney cells while hexanic extract showed the lowest activity. When we compared the protective effect of different extracts, MEAE resulted as the best extract among all the others. However, this extract was less effective (2.4 fold) than quercetin at 200 ppm. AE and MEAE were the most effective in the three evaluated concentrations.

Acute inflammation is characterized by vasodilatation, leukocyte infiltration, inflammation mediators release and edema. With respect to edema (weight) all extracts were different from TPA. However, indomethacin was more effective to reduce edema weight with respect to AF extracts. These results are in accordance to IL-6 and MPO; i.e., all extracts were different from TPA but were less effective than indomethacin to reduce edema. Moreover, CE, HE, MEAE and AE were efficient to reduce ear thickness as indomethacin did. It is important to point out that the topical application of TPA alone elevated edema (thickness and weight) at larger extent than the rest of the extracts. The presence of certain bioactive compounds with recognized potent antioxidant activity as gallic acid, catechin, epicatechin found in KE, ME and MEAE measured by TBARS and DPPH• are close related to the anti-inflammatory activity response. It is known that these antioxidants inhibit the reactive oxygen species generation (e.g., H_2_O_2_) while increasing the scavenging activity [7]. Moreover, the ORAC test and the prostaglandin production (COX) indicated that CE and HE are more successful to scavenge the ROS than the rest of the extracts and to decrease the production of inflammatory cytokines. Likewise, HE reduced the nitrite production from macrophages induced by LPS of *E. coli*. Because cytokines and prostaglandins are part of the mediators responsible of the inflammatory pathways, the damage caused in the cell membranes was inhibited by the presence of lupeol, α-amyrin and β-amyrin, which are compounds that increased the anti-inflammatory response by decreasing the nitrite and the PGE2 levels by inhibition of the iNOS and COX expression, respectively, according to Fernández et al. [31] and Adkins and Kelley [32]. Thus, triterpenes found in CE and HE contributed to exert their anti-inflammatory activity by reducing cytokines such as TNF-α and IL-1β. The mechanism of action of these bioactive compounds is through the modulation of the transcription factor NF-κB [33,34]. The TNF-α expression is consistent with the findings of the protective effect on porcine kidney cells and the immunohistochemistry of TNF-α antibody, since HE was the extract with the lowest production of this pro-inflammatory cytokine. Our results agreed to those reported by Horinouchi et al. [35] who evaluated the ethanolic extracts of flowers from *Combretum leprosum*. In our study MPO activity, IL-6 levels and ear thickness diminished with the topical administration of the extract and no pro-inflammatory activity was observed after the topical application of AF extracts. However, indomethacin decreased ear edema, MPO activity and IL-6 at greater extent than AF extracts. In the same line, Oliveira et al. [8] evaluated the ethanolic extract of aerial parts of *Leonurus sibiricus* found similar results; i.e., reduction in the ear thickness, MPO activity; as well as the concentration of TNF-α and IL-1β. Sánchez et al. [36] and Crispo et al. [37] indicated that functional compounds as flavonoids are responsible of such anti-inflammatory and antioxidant protective actions. Besides the important antioxidant activity, flavonoids interfered with the inflammation process [29]. The inhibition of arachidonic acid (C20:4 n-6) and prostaglandins biosynthesis (i.e., lipoxygenase, phospholipase and cyclooxygenase activities) are included in the processes described for this desirable effect. The metabolic pathway is supported by the investigation of Lin et al. [28] who demonstrated that flavonoids constrain the expression and activation of the pro-inflammatory cytokines, TNF-α and COX.

The results from TBARS assay are in accordance to the previous antioxidant assays. Lipid peroxidation inhibition was dose-dependent and ME succeed among the other extracts using the smallest amount to produce that effect, while AE and HE showed poor inhibition on TBARS production. These results are in line with the information reported by Delgadillo et al. [16] who evaluated AF pods. The different antioxidant activity measured by ORAC and FRAP, as well as the protection against H_2_O_2_-induced damage and lipid peroxidation inhibition, may be the consequence of distinct phytochemicals found on each extract. This might have different and enormous implications on biological responses involved, not only for cellular functioning but also for potential therapeutic applications.

The contrasting activity, measured by ORAC and FRAP assays, also was observed by Ou et al. [38] in the antioxidant characterization of 927 vegetables using these same assays and they concluded that these differences are associated to specific chemical reactions involved in these antioxidant tests. This disparity may be explained by the fact that both assays are based on different reaction mechanisms: ORAC determination is based on hydrogen atom transference (HAT) and FRAP test on single electron transfer (SET) reaction. The HAT mechanism occurs when the oxygen radical abstracts a hydrogen atom from antioxidant while in SET reaction the antioxidant provides an electron to the free radical [39]. FRAP assay measures the ferric reducing ability of a sample, there are no presence of free radicals or oxidants such as occurs in the ORAC assay. So, the antioxidant capacity of an antioxidant against a free radical does not necessarily match its ability to reduce Fe^3+^ to Fe^2+^.

In addition to the antioxidant capacity, the differences in the protection against H_2_O_2_-induced damage and lipid peroxidation inhibition, may be the consequence of distinct phytochemicals found on each extract.

## 4. Materials and Methods

### 4.1. Vegetal Collection and Plant Extracts

AF pods of the present study were collected in Acatlán de Osorio in the state of Puebla in México; located between 18°04′24” and 18°21′30”, north latitude and 97°55′18” and 98°11′24” west longitude. *Acacia farnesiana–*AF (name listed in: http://www.theplantlist.org; accessed on 22 September 2017) pods were registered with an internal identification number (8757) at the herbarium of the Facultad de Estudios Superiores Cuautitlán at the Universidad Nacional Autónoma de México (UNAM). Sampling was performed on aerial parts of AF trees to complete two batches of 20 kg each, which were assessed separately in the subsequent chemical analysis. Pods were completely dried in a drying stove of atmospheric flow at 40 °C for 48 h and grounded using a knife mill to obtain a particle size of 1 mm. The pods of AF were treated individually with different solvents (chloroform, hexane, acetone, methanol, methanol:water (80:20 *v*/*v*) or distilled water) as follows: 200 g of sample were weighed and 500 mL of each solvent were added individually into five Erlenmeyer flasks and then sonicated for 30 min at room temperature. Samples were filtered and concentrated on a rotary evaporator; water bath was set at 40 °C for organic solvents and 60 °C for the aqueous extract. Flasks were washed with the corresponding extractant (10 mL max) and then, the extracts were placed in a vacuum desiccator for a week and stored at 4 °C in amber vials until analyzed. The solvents/aqueous extractions were performed twice.

### 4.2. Total Phenolic Content

Total phenolic content in CE, HE, KE, ME, MEAE and AE of AF pods was determined by the Folin–Ciocalteu colorimetric method described by Singleton et al. [40]. Briefly, 3 mg of each extract were diluted into 1 mL of solvent. Dimethyl sulfoxide (DMSO) was used for HE and CE and distilled water for the rest of the extracts. A half mL of each stock solution was transferred into a volumetric flask and 3% HCl was added to a final volume of 5 mL. From the resulting solution, an aliquot of 100 µL was mixed with a 2 mL of 2% NaCO_3_ solution, letting stand during two minutes. Then, 100 µL of Folin–Ciocalteu reagent (previously diluted 1:1 *v*/*v* with water) were added to the latest solution and incubated at room temperature during 30 min in darkness. Absorbance was measured at 765 nm in a 3.0 mL UV-Quartz cell (Hach Co. cat. 48228-00, Loveland, CO, USA) using a UV spectrophotometric equipment (Beckman, DU-70, Brea, CA, USA). A calibration curve of gallic acid was prepared to estimate the concentration of total polyphenols and results were expressed as mg of gallic acid equivalents (GAE) per 100 g of extract.

### 4.3. UPLC-ESI-Q-oa/TOF-MS Analysis

AF pod extracts were analyzed by Ultra Performance Liquid Chromatography (UPLC) and the chromatographic separations were carried using a Beh Phenyl (2.1 mm × 100 mm, 1.7 mm; Waters, Elstree, UK) analytical column operated at 40 °C. The mobile phase consisted of two solvents: formic acid (0.1%, *v*/*v*) in water (A) and acetonitrile (B). The gradient program was used as follows: 97% A for 1.10 min, followed by multiple gradients from 5% B to 15% B from 1.10 to 4.40 min, holding 15% B for 4.60 min, getting back to the initial conditions (3% B) in 1 min and re-equilibration of the column. Mass spectrometric analyses were performed using a quadrupole time of flight orthogonal accelerated mass spectrometer with electrospray ionization (ESI-Q-oa-TOF-MS, Waters, UK). The solvent flow rate was 0.3 mL min^−1^ with a dry gas temperature of 210 °C, and a dry gas flow of 8.0 L min^−1^. ESI ionization conditions were a capillary voltage of −3.5 and +4.0 kV, and spectra rate of 1 Hz. While, spectra detection was carried out in the negative ion mode and recorded using a scan range *m*/*z* 50–1200 Da using a ramp collision energy of 15–35 V with argon as collision gas. Finally, the identification of the present compounds was determined by using the full mass spectrum and its unique mass fragmentation spectrum and comparing them with the literature, using the NIST, MassBank, Pubchem and mzCloud [https://www.mzcloud.org/] data bases.

### 4.4. Antioxidant Activity

To evaluate the quantitative antioxidant activity, DPPH• (2,2-diphenyl-1-picrylhydrazyl) reagent was employed according to Koren et al. [41]. The antioxidant values of samples were compared with standards of quercetin. To evaluate the peroxyl scavenging capacity by oxygen radical absorbance capacity (ORAC), the methodology of Huang et al. [42] was used. The results are reported as micromoles of Trolox equivalents per gram of extract. Ferric reducing antioxidant power (FRAP) was performed as previously described by Benzie and Strain [43]. The methodology used to evaluate the defense of extracts against oxidative-induced damage was according to Hernández et al. [44] with some modifications. Briefly, culture cells were seeded at a density of 20,000 per well, incubated in 48 well plates for 24 h. Three mg of each extract were diluted into one mL of solvent (3 mg/mL). DMSO was employed for HE and CE and distilled water for the rest of the extracts. Three different dilutions were prepared to obtain final concentrations of 50, 100 and 200 ppm in Dulbecco’s modified eagle medium (DMEM), 300 μL from each solution were added to the plates. In the same well plate, a hydrogen peroxide (H_2_O_2_) solution was added as external oxidant (47 μL, 1 mM) during 2 h. All extracts were evaluated in triplicate.

Lipid peroxidation was evaluated by measuring the thiobarbituric acid reactive substances (TBARS). Cerebral tissue (whole brain) of adult male Wistar rats (200–250 g body weight) was homogenized using phosphate buffer saline (PBS) that was centrifuged (radius of 90 mm) for 10 min at 3000 rpm to yield a pellet, which was discarded. The supernatant protein content was measured using the Lowry et al. [45] methodology and adjusted at 2.666 mg of protein/mL with PBS.

The TBARS quantification was employed according to the method described by Ohkawa et al. [46] with some modifications. Using a bath ice and 1.5 mL micro centrifuge tubes, 375 µL of the supernatant (equivalent to 1 mg of protein) were mixed with 50 µL of EDTA (20 µM in saline). Stock solutions were previously prepared (3 mg/mL) as follows: HE and CE were dissolved in DMSO, and the rest of the extracts in distilled water to get the corresponding dilutions. Then, 25 µL of each extract solution were added to the reaction tube, followed by an incubation for 30 min at 37 °C and shaking. Lipid peroxidation was triggered by adding 50 µL of 100 µM Fe_2_SO_4_ solution and incubated at 37 °C for 1 h under the same conditions described above. TBARS content was determined as follows: 0.5 mL of TBA reagent (0.5% TBA in 0.05 N NaOH and 30% TCA, 1:1 *v*/*v*) was added at each tube and the final suspension was cooled on ice for 10 min, centrifuged (5 min at 12,000 rpm; radius of 90 mm) and heated at 75 °C in a water bath for 30 min. After cooling at room temperature, the absorbance (200 µL) was measured at λ = 540 nm in a microplate reader (Bio-Tek Instruments, Winooski, VT, USA). Concentration of TBARS was calculated by interpolation in a standard curve of tetramthoxypropane [47]. Results were expressed as nmol of TBARS per mg of protein. Quercetin was used as a positive standard. The inhibition ratio (IR [%]) was calculated using the following formula IR = (C − E/C) × 100, where C is the absorbance of control and E is the absorbance of the sample. These values were plotted against the log of the concentrations of individual extracts, and a decrease of 50% in peroxidation was defined as the EC_50_.

### 4.5. Anti-Inflammatory Activity

#### 4.5.1. Animal Experimental Set Up

The experimental animals (male CD-1 mice; average live weight 25–30 g) were obtained from the Universidad Autónoma Metropolitana-Xochimilco (UPEAL-Bioterio, DCBS.BIOT.390.16), Mexico City. Animals (eight groups with six animals each one), were housed at 22 ± 4 °C with 12/12 h light-darkness cycles and relative humidity of 40–70% at the Instituto de Química UNAM. Food and plain water were provided ad libitum. All experimental procedures followed the recommendations of the Mexican Federal Regulation (NOM-062-ZOO-1999). Protocols for maintenance, sample collection and euthanasia were reviewed and approved by the internal committee for the care and the use of lab animals of the Instituto de Química at UNAM (CICUAL-IQ-004-17).

#### 4.5.2. Ear Edema Essay in CD-1 Mice Induced with 12-*O*-tetradecanoylphorbol Acetate (TPA)

This essay was performed according to Del-Ángel et al. [48] with some modifications. 20 µL of solutions of the six extracts at a concentration of 3 mg/ear and indomethacin as standard (1 mg/ear) were used. To facilitate the miscibility of the samples, for CE and HE extracts, chloroform was used as vehicle while methanol was used for KE, ME, MEAE and AE extracts. For the standard (indomethacin), the vehicle solution used was a mixture of acetone and methanol (1:1). Solutions were homogenized with a vortex during 30 s. On other hand, the rodents were previously anesthetized with an intraperitoneal injection of sodium pentobarbital (63 mg/kg) to produce general anesthesia during 20 to 50 min. Palpebral, patellar and ocular reflexes were monitored to confirm adequate anesthesia of mice. Subsequently 10 µL of ethanolic solution of TPA (250 µg/mL) were applied on top of the right ear of the mice. Ten minutes later, on top of the same ear (right ear) either 20 µL of the extracts or indomethacin were applied. On top of the left ear (control ear) of mice, 10 μL of ethanol plus 20 μL of the corresponding vehicle solution were applied. After these procedures, mice were located into a sand-dust bedded plastic box over a heater (26–28 °C) to avoid thermic stress during the recovery period from anesthesia. Once mice were fully recovered, they were transferred to a new plastic box and were monitored during four hours. After this period, the euthanasia was performed in a CO_2_ chamber. Round biopsies of 7 mm of diameter in both ears from each mouse were taken using a straight punch tool (ACE surgical supply CO., Inc.). Biopsies were weighed and the edema inhibition (%) was obtained using the following formula: percentage of inhibition = (C−E/C) × 100; where C = was the edema in the positive control group (TPA application alone); E = edema in the experimental groups treated with either AF extracts or indomethacin [48].

#### 4.5.3. Oxidative Enzyme Myeloperoxidase Assay (MPO)

Ears biopsies (preserved at −80 °C) coming from the TPA-induced edema test were used for this assessment. The methodology used for this essay was carried out in agreement with the reports of Suzuki et al. [49] and Bradley et al. [50]. The inhibition of the MPO activity was calculated using the formula: Percentage of MPO inhibition = ODC−ODE/ODC × 100; where ODC = optical density at 450 nm of control group (ethanol plus extracts or standard) and ODE = optical density at 450 nm of experimental groups (TPA plus extracts or standard).

#### 4.5.4. Histological Analysis of Ear

The employed methodology for this essay was carried out according to previous reports [49,50]. For this analysis, we used complete ears of CD-1 mouse from the TPA-induced edema test. The ears were immediately fixed in 10% formaldehyde. A train of dehydration was used; i.e., ear samples were sinked successively during 30 min into each of the following solvents: ethanol (50%), ethanol (60%), ethanol (70%), ethanol (80%), ethanol (96%), ethanol (100%) and xylol (100%). Once the biological sample was dry, were embedded in paraffin (60 °C) and cut into sections (4 μm slices) and stained with hematoxylin and eosin. The tissues were observed under a microscope (Leica DM750 Wetzlar, Hesse, Germany), photographed using a digital camera (Leica DMC2900) and processed with the imaging software Leica LAS Core V4.5 (Bufallo Grove, IL, USA).

#### 4.5.5. Immunohistochemistry

The assay was performed according to Leal-Díaz et al. [51] and Méndez-Flores et al. [52] with some modifications. Tumor necrosis factor (TNF)-α expressing cells were determined in 4 μm thick sections of available formalin-fixed paraffin embedded tissue. Endogenous peroxidase and binding of nonspecific proteins were blocked with 3% H_2_O_2_ and 10% of normal donkey serum (ABC Staining System; Santa Cruz Biotechnology, Dallas, TX, USA), respectively. Tissues were incubated with rabbit monoclonal anti-rat TNF-α antibody (Merck Millipore, Billerica, MA, USA) diluted to 10 µg/mL and let stand for 18 h at 4 °C. Binding was identified with biotinylated secondary antibody and horseradish peroxidase-(HRP)-streptavidin (ABC Staining System; Santa Cruz Biotechnology). Slides were incubated with substrate 3,3’-diaminobenzidine (DAB) (Sigma-Aldrich, St. Louis. MO, USA) for 10 min. The sections were counterstained with hematoxylin, dehydrated and mounted in resin. Negative control staining was performed with normal donkey serum diluted 1:100, instead of primary antibody, and the immunohistochemistry (IHC) universal negative control reagent specifically designed to work with rabbit, mouse, and goat antibodies (IHC universal negative control reagent, Enzo Life Sciences, Inc., Farmingdale, NY, USA, ADI-950-231). The reactive blank was incubated with phosphate buffer saline-egg albumin (Sigma-Aldrich) instead of the primary antibody. Both controls excluded nonspecific staining or endogenous enzymatic activities. The tissues were observed, photographed and processed as above described.

#### 4.5.6. Quantitation of IL-1β, IL-6, IL-10 and Tumor Necrosis Factor α (TNF-α) from Ear Extracts

Ear punches were homogenized with 500 mL of cold lysis buffer (Merck), centrifuged and stored at −80 °C until analyzed. Interleukin (IL) IL-1β, IL-6, IL-10 and the TNF-α from ears biopsies coming from the TPA-induced edema test were analyzed using a 96-well plate of Milliplex MAP kit, with a mouse cytokine/chemokine multiplex magnetic bead panel (Cat. MCYTOMAG-70K, Merck). Samples were analyzed in a MAGPIX luminometer and the concentration of each metabolite calculated with a 5-parameter polynomial standard curves ranging from 0.65 to 10,000 pg/mL using the xPonent 4.2 software (Luminex Corporation, Austin, TX, USA). All samples from each treatment were analyzed simultaneously to minimize error.

### 4.6. In Vitro COX Inhibition and Nitrite Assay

The first in vitro assay was performed in a COX inhibitor screening kit (Cayman Chemical Company, Ann Arbor, MI, USA) according to Del-Ángel et al. [48] as well as the in vitro nitrite assay, which include: (1) the culture of RAW 264.7 cells; (2) the nitrite assay where cells were treated with lipopolysaccharides (*E. coli*, serotype 055 B5) to observe production of nitric oxide (NO); and (3) the cell viability assay by the incorporation of 3-(4,5-dimethylthiazol-2-yl)-2,5-diphenyl tetrazolium bromide (MTT) by RAW 264.7 cells.

### 4.7. Statistical Analysis

Non-parametric Kruskal–Wallis test was used to compare groups while U de Mann-Whitney test was employed to observe differences among peers (α = 0.05). SPSS statistic software was used (18th V. IBM Corp, Armonk, NY, USA).

## 5. Conclusions

*Acacia farnesiana* pod extracts can be effective as antioxidant and as topical anti-inflammatory agents. This investigation helps to support the traditional use of this vegetal resource as alternative medicine. No clear evidence was observed among the six extracts to identify the best extractant to endorse both antioxidant and anti-inflammatory properties. Therefore, it is necessary to continue the research in this vegetal resource to elucidate the molecular mechanism of these activities which make this plant a real opportunity to consider as an alternative source of such compounds. Additionally, the same pod extracts are under evaluation to elucidate their capacity to act as antitubercular and antidysenteric agent. The inclusion of AF pods in ruminant diets will be conducted to observe the modification of milk in terms of bioactive constituents present for human consumption.

## Figures and Tables

**Table 1:** Compounds identified in six extracts from *Acacia farnesiana* (AF) pods.

Peak	Rt (min)	[M − H]^−^ (*m*/*z*)	Molecular Formula	Dominant Ion Fragment	Assignment	Reference
**Chloroformic**						
1	0.866	387.0918	-	341.1011 (100)	Unknow	-
2	0.935	331.0602	C_13_H_16_O_10_	271.0561 (100)	Galloyl glucose isomer 1	[19]
3	1.826	169.0484	C_7_H_6_O_5_	125.0678 (100)	Gallic acid	[20]
4	1.912	331.0612	C_13_H_16_O_10_	271.0566 (100)	Galloyl glucose isomer 2	[19]
5	2.169	331.0610	C_13_H_16_O_10_	271.0572 (100)	Galloyl glucose isomer 3	[19]
6	2.512	493.0616	-	271.0562 (100)	Unknow	-
7	4.673	183.0608	C_8_H_8_O	168.9363 (100)	Methyl gallate	[21]
8	4.776	483.0230	C_20_H_20_O_14_	-	Digalloyl glucose isomer 1	[22]
9	4.982	483.0224	C_20_H_20_O_14_	-	Digalloyl glucose isomer 2	[22]
**Hexanic**						
1	0.866	387.0918	-	341.1011 (100)	Unknow	-
2	0.935	331.0602	C_13_H_16_O_10_	271.0561 (100)	Galloyl glucose isomer 1	[19]
3	1.826	169.0484	C_7_H_6_O_5_	125.0678 (100)	Gallic acid	[20]
4	1.912	331.0612	C_13_H_16_O_10_	271.0566 (100)	Galloyl glucose isomer 2	[19]
5	2.169	331.0610	C_13_H_16_O_10_	271.0572 (100)	Galloyl glucose isomer 3	[19]
6	2.512	493.0616	-	271.0562 (100)	Unknow	-
7	4.673	183.0608	C_8_H_8_O	168.9363 (100)	Methyl gallate	[21]
8	4.776	483.0230	C_20_H_20_O_14_	-	Digalloyl glucose isomer 1	[22]
9	4.982	483.0224	C_20_H_20_O_14_	-	Digalloyl glucose isomer 2	[22]
**Ketonic**						
1	0.917	331.0600	C_13_H_16_O_10_	271.0560 (45) 211.0483 (1)	Galloyl glucose isomer 1	[19]
2	1.826	331.0605	C_13_H_16_O_10_	271.0565 (100)	Galloyl glucose isomer 2	[19]
3	2.084	331.0597	C_13_H_16_O_10_	271.0559 (100)	Galloyl glucose isomer 3	[19]
4	2.392	493.0606	-	271.0557 (17) 313.0538 (1) 169.0465 (1)	Unknow	-
5	4.279	483.0223	C_20_H_20_O_14_	183.0624 (100)	Digalloyl glucose isomer 1	[23]
6	4.588	183.0610	C_8_H_8_O	168.9363 (100)	Methyl gallate	[21]
7	4.691	483.0228	C_20_H_20_O_14_	271.0562 (15) 211.0498 (8) 169.0481 (49) 125.0660 (1)	Digalloyl glucose isomer 2	[22]
8	4.914	483.0219	C_20_H_20_O_14_	271.0562 (36) 211.0494 (3) 169.0481 (28) 125.0646 (1)	Digalloyl glucose isomer 3	[22]
9	5.051	483.0226	C_20_H_20_O_14_	-	Digalloyl glucose isomer 4	[22]
10	5.651	401.0812	-	-	Unknow	-
**Methanolic**						
1	0.832	331.0601	C_13_H_16_O10	271.0561 (40) 169.0482 (1) 133.0557 (1)	Galloyl glucose isomer 1	[19]
2	1.072	331.0606	C_13_H_16_O_10_	271.0565 (100)	Galloyl glucose isomer 2	[19]
3	1.603	483.0223	C_20_H_20_O_14_	271.0487 (100)	Digalloyl glucose isomer 1	[19]
4	1.912	483.0223	C_20_H_20_O_14_	271.0566 (100)	Digalloyl glucose isomer 2	[19]
5	2.581	183.0606	C_8_H_8_O	168.9363 (100)	Methyl gallate	[21]
**Methanolic: Aqueous**						
1	0.9	387.0913	-	377.0659 (40) 341.1000 (18)	Unknow	-
2	1.02	331.0603	C_13_H_16_O_10_	271.0562 (100)	Galloyl glucose isomer 1	[19]
3	1.946	169.0483	C_7_H_6_O_5_	125.0661 (100)	Gallic acid	[19]
4	2.118	331.0606	C_13_H_16_O_10_	271.0565 (100)	Galloyl glucose isomer 2	[19]
5	2.41	331.0611	C_13_H_16_O_10_	271.0569 (100)	Galloyl glucose isomer 3	[19]
6	2.821	493.0619	-	271.0559 (100)	Unknow	-
7	4.965	183.0604	C_8_H_8_O	168.9363 (100)	Methyl gallate	[21]
8	5.171	483.0225	C_20_H_20_O_14_	271.0564 (100)	Digalloyl glucose isomer 1	[22]
**Aqueous**						
1	0.8666	195.0799	-	165.0759 (100)	Hydroxytyrosol acetate	[24]
2	1.02	133.0546	-	115.0463 (100)	unknow	-
3	1.26	191.0494	C_7_H_12_O_6_	111.0524 (100)	Quinic acid	[25]
4	1.398	295.0470	C_13_H_12_O_8_	-	Caffeoylmalic acid	[26]
5	1.929	169.0478	C_7_H_6_O_5_	125.0657 (100)	Gallic acid	[23]
6	4.879	183.0609	C_8_H_8_O	168.9363 (100)	Methyl gallate	[21]

## Supplementary Materials

Supplementary Materials are available online. Figure S1: Chromatographic spectra of AF extracts.

## References

[1] Halliwell B. (2012). Free radicals and antioxidants: Updating a personal view. Nutr. Rev..

[2] Stevenson D.E., Hurst R.D. (2007). Polyphenolic phytochemicals–just antioxidants or much more?. Cell. Mol. Life Sci..

[3] Correa L.B., Pádua T.A., Seito L.N., Costa T.E.M.M., Silva M.A., Candéa A.L.P., Rosas E.C., Henriques M.G. (2016). Anti-inflammatory effect of methyl gallate on experimental arthritis: Inhibition of neutrophil recruitment, production of inflammatory mediators, and activation of macrophages. J. Nat. Prod..

[4] Hernández-Valle E., Herrera-Ruiz M., Salgado G., Zamilpa A., Ocampo M., Aparicio A., Tortoriello J., Jiménez-Ferrer E. (2014). Anti-inflammatory effect of 3-*O*-[(6′-*O*-Palmitoyl)-β-d-glucopyranosyl sitosterol] from Agave angustifolia on ear edema in mice. Molecules.

[5] Hou X.L., Tong Q., Wang W.Q., Shi C.Y., Xiong W., Chen J., Liu X., Fang J.G. (2015). Suppression of inflammatory responses by dihydromyricetin, a flavonoid from Ampelopsis grossedentata, via inhibiting the activation of NF-κB and MAPK signaling pathways. J. Nat. Prod..

[6] Gasparrini M., Giampieri F., Forbes-Hernandez T.Y., Afrin S., Cianciosi D., Reboredo-Rodriguez P., Valera-Lopez A., Zhang J.J., Quiles L.J., Mezzetti B. (2018). Strawberry extracts efficiently counteract inflammatory stress induced by the endotoxin lipopolysaccharide in Human Dermal Fibroblast. Food Chem. Toxicol..

[7] Domínguez M., Nieto A., Marin J.C., Keck A.S., Jeffery E., Céspedes C.L. (2005). Antioxidant activities of extracts from *Barkleyanthus salicifolius* (Asteraceae) and *Penstemon gentianoides* (Scrophulariaceae). J. Agri. Food Chem..

[8] Oliveira A.S., Cercato L.M., de Santana Souza M.T., Melo A.J.O., Lima B.D.S., Duarte M.C., Araujo A.A.S., de Oliveira e Silva A.M., Camargo E.A. (2017). The ethanol extract of *Leonurus sibiricus* L. induces antioxidant, antinociceptive and topical anti-inflammatory effects. J. Ethnopharmacol..

[9] Rauh L.K., Horinouchi C.D.S., Loddi A.M.V., Pietrovski E.F., Neris R., Souza-Fonseca-Guimarães F., Buchi D.F., Biavatti M.W., Otuki M.F., Cabrini D.A. (2011). Effectiveness of *Vernonia scorpioides* ethanolic extract against skin inflammatory processes. J. Ethnopharmacol..

[10] Abdallah H.M., Esmat A. (2017). Antioxidant and anti-inflammatory activities of the major phenolics from Zygophyllum simplex L. J. Ethnopharmacol..

[11] Fortunato L.R., Alves C.D.F., Teixeira M.M., Rogerio A.P. (2012). Quercetin: A flavonoid with the potential to treat asthma. Braz. J. Pharm. Sci..

[12] Sathya A., Siddhuraju P. (2013). Protective effect of bark and empty pod extracts from Acacia auriculiformis against paracetamol intoxicated liver injury and alloxan induced type II diabetes. Food Chem. Toxicol..

[13] Hernández G.E. (2017). Aislamiento, caracterización estructural y determinación de las propiedades antibacterianas de los constituyentes de los frutos de *Acacia farnesiana*. Master’s Thesis.

[14] Fernandez P.M.S., Villano D., Troncoso A.M., Garcia P.M.C. (2008). Antioxidant activity of phenolic compounds: From in vitro results to in vivo evidence. Crit. Rev. Food Sci. Nutr..

[15] Cuchillo H.M., Puga D.C., Wrage-Mönning N., Espinosa M.J.G., Montaño B.S., Navarro-Ocaña A., Ledesma J.A., Diaz M.M., Pérez-Gil R.F. (2013). Chemical composition, antioxidant activity and bioactive compounds of vegetation species ingested by goats on semiarid rangelands. J. Anim. Feed Sci..

[16] Delgadillo P.C., Cuchillo H.M., Espinosa M.J.G., Medina C.O., Molina J.E., Díaz M.M., Álvarez I.M.A., Ledesma S.J.A., Pedraza-Chaverri J. (2015). Antioxidant activity and protection against oxidative-induced damage of *Acacia shaffneri* and *Acacia farnesiana* pods extracts: In vitro and in vivo assays. BMC Complement. Altern. Med..

[17] Akdis M., Burgler S., Crameri R., Eiwegger T., Fujita H., Gomez E., Klunker S., Meyer N., O’Mahony L., Palomares O. (2011). Interleukins, from 1 to 37, and interferon-γ: Receptors, functions, and roles in diseases. J. Allergy Clin. Immunol..

[18] García J.S.A., Verde M.J.S., Heredia L.N. (2001). Traditional uses and scientific knowledge of medicinal plants from Mexico and Central America. J. Herbs Spices Med. Plants.

[19] Bae S., Kim S.Y., Do M.H., Lee C.H., Song Y.J. (2017). 1,2,3,4,6-Penta-*O*-galloyl-ß-d-glucose, a bioactive compound in Elaeocarpus sylvestris extract, inhibits varicella-zoster virus replication. Antivir. Res..

[20] Ambigaipalan P., de Camargo A.C., Shahidi F. (2017). Identification of phenolic antioxidants and bioactives of pomegranate seeds following juice extraction using HPLC-DAD-ESI-MSn. Food Chem..

[21] Ma C., Zhao X., Wang P., Jia P., Zhao X., Xiao C., Zheng X. (2018). Metabolite characterization of Penta-*O*-galloyl-beta-d-glucose in rat biofluids by HPLC-QTOF-MS. Chin. Herb. Med..

[22] Li J., Kuang G., Chen X., Zeng R. (2016). Identification of chemical composition of leaves and flowers from Paeonia rockii by UHPLC-Q-Exactive Orbitrap HRMS. Molecules.

[23] Slatnar A., Mikulic-Petkovsek M., Stampar F., Veberic R., Solar A. (2015). Identification and quantification of phenolic compounds in kernels, oil and bagasse pellets of common walnut (*Juglans regia* L.). Food Res. Int..

[24] Tasioula-Margari M., Tsabolatidou E. (2015). Extraction, separation, and identification of phenolic ompounds in virgin olive oil by HPLC-DAD and HPLC-MS. Antioxidants.

[25] Llorent-Martínez E.J., Spínola V., Castilho P.C. (2017). Phenolic profiles of Lauraceae plant species endemic to Laurisilva forest: A chemotaxonomic survey. Ind. Crops Prod..

[26] Spínola V., Pinto J., Castilho P.C. (2015). Identification and quantification of phenolic compounds of selected fruits from Madeira Island by HPLC-DAD–ESI-MSn and screening for their antioxidant activity. Food Chem..

[27] Salem M.M., Davidorf H.F., Abdel-Rahman H.M. (2011). In vitro anti-uveal melanoma activity pf phenols compounds fron the Egyptian medicianla plant *Acacia nilotica*. Fitoterapia.

[28] Lin A.S., Lin C.R., Du Y.C., Lübken T., Chiang M.Y., Chen I.H., Wu C.C., Hwang T.L., Chen S.L., Yen M.H. (2009). Acasiane A and B and farnesirane A and B, diterpene derivatives from the roots of Acacia farnesiana. Planta Med..

[29] Yi J., Zhu R., Wu J., Wu J., Xia W., Zhu L., Jiang W., Xiang S., Tan Z. (2016). In vivo protective effect of betulinic acid on dexamethasone induced thymocyte apoptosis by reducing oxidative stress. Pharmacol. Rep..

[30] Karoune S., Falleh H., Kechebar M.S.A., Halis Y., Mkadmini K., Belhamra M., Rahmoune C., Ksouri R. (2015). Evaluation of antioxidant activities of the edible and medicinal Acacia albida organs related to phenolic compounds. Nat. Prod. Res..

[31] Fernández M.A., de las Heras B., Garcia M.D., Sáenz M.T., Villar A. (2001). New insights into the mechanism of action of the anti-inflammatory triterpene lupeol. J. Pharm. Pharmacol..

[32] Adkins Y., Kelley D.S. (2010). Mechanisms underlying the cardioprotective effects of omega-3 polyunsaturated fatty acids. J. Nutr. Biochem..

[33] Endo J., Arita M. (2016). Cardioprotective mechanism of omega-3 polyunsaturated fatty acids. J. Cardiol..

[34] Romero E.A., Maldonado M.A., González C.J., Bahena S.M., Garduño R.M.L., Rodríguez L.V., Alvarez L. (2016). Anti-inflammatory and antioxidative effects of six pentacyclic triterpenes isolated from the Mexican copal resin of Bursera copallifera. BMC Complement. Altern. Med..

[35] Horinouchi C.D., Mendes D.A., Soley Bda S., Pietrovski E.F., Facundo V.A., Santos A.R.S., Cabrini D.A., Otuki M.F. (2013). Combretum leprosum Mart. (Combretaceae): Potential as an antiproliferative and anti-inflammatory agent. J. Ethnopharmacol..

[36] Sánchez E., Heredia N., Camacho C.M.R., García S. (2013). Isolation, characterization and mode of antimicrobial action against Vibrio cholerae of methyl gallate isolated from *Acacia farnesiana*. J. App. Microbiol..

[37] Crispo J.A.G., Piché M., Ansell D.R., Eibl J.K., Tai I.T., Kumar A., Ross G.M., Tai T.C. (2010). Protective effects of methyl gallate on H_2_O_2_-induced apoptosis in PC12 cells. Biochem. Biophys. Res. Commun..

[38] Ou B., Huang D., Hampsch-Woodill M., Flanagan J.A., Deemer E.K. (2002). Analysis of antioxidant activities of common vegetables employing oxygen radical absorbance capacity (ORAC) and ferric reducing antioxidant power (FRAP) assays: A comparative study. J. Agric. Food Chem..

[39] Liang N., Kitts D.D. (2014). Antioxidant property of coffee components: Assessment of methods that define mechanisms of action. Molecules.

[40] Singleton V.L., Orthofer R., Lamuela-Raventós R.M. (1999). Analysis of total phenols and other oxidation substrates and antioxidants by means of Folin-ciocalteu reagent. Methods Enzymol..

[41] Koren E., Kohen R., Ginsburg I. (2010). Polyphenols enhance total oxidant-scavenging capacities of human blood by binding to red blood cells. Exp. Biol. Med..

[42] Huang D., Ou B., Hampsch-Woodill M., Flanagan J.A., Prior R.L. (2002). High-throughput assay of oxygen radical absorbance capacity (ORAC) using a multichannel liquid handling system coupled with a microplate fluorescence reader in 96-well format. J. Agric. Food Chem..

[43] Benzie I.F.F., Strain J.J. (1996). The ferric reducing ability of plasma (FRAP) as a measure of “antioxidant power”: The FRAP assay. Anal. Biochem..

[44] Hernández F.K., Cárdenas R.N., Pedraza C.J., Massieu L. (2008). Calcium-dependent production of reactive oxygen species is involved in neuronal damage induced during glycolysis inhibition in cultured hippocampal neurons. J. Neurosci. Res..

[45] Lowry O.H., Rosebrough N.J., Farr A.L., Randall R.J. (1951). Protein measurement with the Folin phenol reagent. J. Biol. Chem..

[46] Ohkawa H., Ohishi N., Yagi K. (1979). Assay for lipid peroxides in animal tissues by thiobarbituric acid reaction. Anal. Biochem..

[47] Esterbauer H., Cheeseman K.H. (1990). Determination of aldehydic lipid peroxidation products: Malonaldehyde and 4-hydroxynonenal. Methods Enzymol..

[48] Del-Ángel M., Nieto A., Ramírez-Apan T., Delgado G. (2015). Anti-inflammatory effect of natural and semi-synthetic phthalides. Eur. J. Pharmacol..

[49] Suzuki K., Ota H., Sasagawa S., Sakatani T., Fujikura T. (1983). Assay method for myeloperoxidase in human polymorphonuclear leukocytes. Anal. Biochem..

[50] Bradley P.P., Priebat D.A., Christensen R.D., Rothstein G. (1982). Measurement of cutaneous inflammation: Estimation of neutrophil content with an enzyme marker. J. Investig. Dermatol..

[51] Leal-Díaz A.M., Noriega L.G., Torre-Villalvazo I., Torres N., Alemán-Escondrillas G., López-Romero P., Sánchez-Tapia M., Aguilar-López M., Furuzawa-Carballeda J., Velázquez-Villegas L.A. (2016). Aguamiel concentrate from Agave salmiana and its extracted saponins attenuated obesity and hepatic steatosis and increased Akkermansia muciniphila in C57BL/6 mice. Sci. Rep..

[52] Méndez-Flores S., Hernandez-Molina G., Enriquez A.B., Faz-Muñoz D., Esquivel Y., Pacheco-Molina C., Furuzawa-Carballeda J. (2016). Cytokines and effector/regulatory cells characterization in the physiopathology of cutaneous lupus erythematous: A cross-sectional study. Mediat. Inflamm..

