# Supplementary material for: Phenolic Compounds in Organic and Aqueous Extracts from Acacia farnesiana Pods Analyzed by ULPS-ESI-Q-oa/TOF-MS. In Vitro Antioxidant Activity and Anti-Inflammatory Response in CD-1 Mice
Source: Molecules. 2018 Sep 18;23(9):2386. doi: 10.3390/molecules23092386 (PMC6225385; doi:10.3390/molecules23092386)
Supplement: Supplementary file 1 [file molecules-23-02386-s001.zip › molecules-353840-SI.doc]

Supplementary material

1) Representative chromatogram of chloroformic extract of *Acacia farnesiana* pods by UPLC-ESI-Q-oa-/TOF-MS

2) Representative chromatogram of hexanic extract of *Acacia farnesiana* pods by UPLC-ESI-Q-oa-/TOF-MS

3) Representative chromatogram of ketonic extract of *Acacia farnesiana* pods by UPLC-ESI-Q-oa-/TOF-MS

4) Representative chromatogram of methanolic extract of *Acacia farnesiana* pods by UPLC-ESI-Q-oa-/TOF-MS

5) Representative chromatogram of methanolic:aqueous extract of *Acacia farnesiana* pods by UPLC-ESI-Q-oa-/TOF-MS

6) Representative chromatogram of aqueous extract of *Acacia farnesiana* pods by UPLC-ESI-Q-oa-/TOF-MS
